# Prefrontal cortex structural and developmental associations with callous-unemotional traits and aggression

**DOI:** 10.1038/s41598-024-54481-3

**Published:** 2024-02-19

**Authors:** Nathan Hostetler, Tamara P. Tavares, Mary B. Ritchie, Lindsay D. Oliver, Vanessa V. Chen, Steven Greening, Elizabeth C. Finger, Derek G. V. Mitchell

**Affiliations:** 1 Brain and Mind Institute, Western Interdisciplinary Research Building, Room 3190, Western University, London, ON N6A 5B7 Canada; 2 Campbell Family Mental Health Research Institute, Centre for Addiction and Mental Health, Toronto, ON Canada; 3 Department of Psychology, University of Manitoba, Winnipeg, MB Canada; 4 Robarts Institute, Western University, 100 Perth Drive, London, ON Canada; 5 Lawson Health Research Institute, 268 Grosvenor Street, London, ON Canada; 6 Parkwood Institute, St. Josephs Health Care, London, ON Canada; 7 Neuroscience and Mental Health Program, The Hospital for Sick Children, Toronto, ON Canada; 8 Department of Psychology, Western University, London, ON Canada; 9 Department of Psychiatry, Western University, London, ON Canada; 10 Department of Anatomy & Cell Biology, Western University, London, ON Canada

**Keywords:** Cognitive neuroscience, Human behaviour, Development of the nervous system

## Abstract

Youths with high levels of callous-unemotional (CU) traits and aggression are at an increased risk for developing antisocial behaviours into adulthood. In this population, neurostructural grey matter abnormalities have been observed in the prefrontal cortex. However, the directionality of these associations is inconsistent, prompting some to suggest they may vary across development. Although similar neurodevelopmental patterns have been observed for other disorders featuring emotional and behavioural dysregulation, few studies have tested this hypothesis for CU traits, and particularly not for aggression subtypes. The current study sought to examine grey matter correlates of CU traits and aggression (including its subtypes), and then determine whether these associations varied by age. Fifty-four youths (10–19 years old) who were characterized for CU traits and aggression underwent MRI. Grey matter volume and surface area within the anterior cingulate cortex was positively associated with CU traits. The correlation between CU traits and medial orbitofrontal cortex (mOFC) volume varied significantly as a function of age, as did the correlation between reactive aggression and mOFC surface area. These associations became more positive with age. There were no significant findings for proactive/total aggression. Results are interpreted considering the potential for delayed cortical maturation in youths with high CU traits/aggression.

## Introduction

Globally, 1.6 million people die each year as a result of violence^1^. While violence is a complex phenomenon with numerous causes, there are a variety of risk factors demarcating the most severe and persistent forms. Early life aggression is one such risk factor, which is predictive of heightened levels of violent offending in adulthood^2^. Often, those displaying this early pattern of aggression are diagnosed with disruptive behavioural disorders, namely oppositional defiant disorder (ODD) or conduct disorder (CD). Individuals with such disorders can be further differentiated by their levels of callous-unemotional (CU) traits^3^. These traits include reduced empathy, a lack of guilt, low concern for one’s own performance, and shallow affect^3^. Youths with elevated levels of CU traits commit more crime^4^, are more violent^5^, and re-offend at higher rates than those without such traits^6^. CU traits are also more closely associated with youth aggression than traditional measures of empathy^7^, and early-adolescent CU traits are predictive of criminal offending in adulthood^8^. In fact, CU traits and aggression in youth are developmental risk factors for adult psychopathy^9^, which has proven to be challenging to treat^10,11^. However, interventions designed specifically for youths with high CU traits show promise when delivered in early childhood^12^. While high levels of both CU traits and aggression are associated with problematic behaviours, it is important to note that both of these features are present along a spectrum across many DSM-5 diagnoses and amongst healthy individuals^13^; consequently, there are thought to be advantages in considering the biological bases for symptoms trans-diagnostically^14,15^. To help inform future treatments that may target symptoms that appear across diagnoses, it is important to consider the neurobiological correlates of both aggression and CU traits across a range of both clinical populations and in typically developing groups.

With respect to prefrontal cortex structure, elevated levels of CU traits (irrespective of aggression levels) in youth have been associated with grey matter abnormalities in the medial orbitofrontal cortex (mOFC), anterior cingulate cortex (ACC), and ventrolateral prefrontal cortex (vlPFC)^16–22^. However, the empirical picture thus far is complex, with inconsistencies in the directionality of these associations. Specifically, in the mOFC and ACC, the existing literature suggests both positive^16,19^ and negative^18,20,21^ morphometric associations with CU traits. Similarly, in the vlPFC, studies have found both negative^17,18^ and positive^16,22^ grey matter morphometric associations with CU traits.

Another early childhood problem associated with persistent violence and criminality into adulthood is aggression^23^. As is observed with CU traits, the association between prefrontal grey matter morphometrics and youth aggression has proven to be inconsistent. For example, total aggression, defined as a summary score from the caregiver-reported Reactive and Proactive Aggression Questionnaire, has been associated with increased volume in the orbitofrontal cortex (OFC)^24^. Contrarily, reduced OFC grey matter volume has been associated with disruptive behavioural disorders, which feature increased rates of aggression^25^.

The empirical picture with respect to aggression is further complicated by the fact that it is characterized as having at least two subtypes^26^. Reactive aggression is elicited by frustration or perceived threat, occurs without regard for a potential goal, and is generally associated with high emotional arousal. Proactive aggression is highly goal-directed, usually aimed at material gain, and need not be accompanied by high emotional arousal^27^. Whereas youths with ODD/CD and high CU traits are at an increased risk for both reactive and proactive aggression, those with low CU traits typically show only reactive aggression^9^. Importantly, youths who exhibit proactive aggression are at the highest risk for later criminality^28^. As such, similar to CU traits, those that exhibit proactive aggression may represent a subgroup of youths with particularly problematic behavioural profiles.

While the association between prefrontal cortical structures and CU traits/aggression is not consistent across studies, the contribution of abnormalities in these areas to the development of these features is predicted by prominent neurocognitive models. Specifically, mOFC and ACC are thought to play a role in empathic concern and moral judgment^29^ by way of encoding the aggressor’s victims’ distress as aversive^30^. This representation of distress is thought to support normal social learning, thereby reducing levels of both CU traits and proactive aggression^31^. More recently, it has been suggested that medial prefrontal cortical areas may also play a role in regulating reactive aggression via computations associated with representing reinforcement expectancies to guide response selection^32^. In contrast, vlPFC is thought to play a role in response inhibition^33^, top-down inhibition of brainstem “fight-or-flight” responses^31^, and the regulation of irritability and frustration^30^. Each of these functions is thought to reduce the risk of reactive aggression. However, these findings are complicated by work implicating the vlPFC in emotional empathy, which is thought to reduce the risk of proactive aggression^34^. Importantly, despite the neurocognitive work suggesting that these prefrontal regions may be differentially associated with proactive and reactive aggression^30,35^, few studies have separately examined the structural correlates of proactive and reactive aggression in youths (for notable exceptions, see^24,25,36^). Considering the directional inconsistencies in the structural associations to these features, it is noteworthy that an emerging stream of research implicates prefrontal cortical maturational abnormalities in a number of conditions featuring emotional and behavioural dysregulation such as disruptive behavioural disorders^37^ and ADHD^38,39^. Thus, correlations between these conditions and morphometrics may change across development and with age. Consequently, the observed inconsistencies in the literature regarding CU traits and aggression may be due in part to the influence of development on the neuroanatomical corelates of these traits (e.g.,^16,19,40^). Indeed, some researchers have suggested that the structural abnormalities associated with CU traits and aggression may reflect abnormalities in cortical maturation and synaptic pruning^16,17^.

These findings regarding cortical maturational deficits have also been corroborated with dimensional measures. Specifically, externalizing behaviours have been linked longitudinally with reduced cortical thinning in childhood^41^. Relating to CU traits, Sumich et al.^40^ found that N200 event-related potential amplitude, which characteristically decreases as the cortex matures, was positively associated with CU traits after covarying for age. This is consistent with the idea that, at a given age, youths with high levels of CU traits have undergone less cortical maturation than typically-developing youths^40^. In terms of longitudinal structural evidence pertaining to CU traits, Tyborowska et al.^19^ found that CU traits are associated with smaller developmental reductions in ACC grey matter volume during adolescence. Complementing these findings, higher levels of prosociality have been shown to be associated with greater prefrontal cortical thinning in adolescence^42^. Notably, these findings relating to CU traits and prosocial behaviours display the same developmental pattern seen in the longitudinal investigations of externalizing behaviours^41^ and conduct problems^39^. Thus, the suggestion from these studies is that larger grey matter maturational reductions, especially in prefrontal areas involved in social cognition, are associated with greater prosocial development. Conversely, smaller grey matter maturational reductions are associated with fewer prosocial behaviours^42^, higher CU traits^19^, more externalizing behaviours^41^, and greater conduct problem severity^39^. These developmental abnormalities may result in structural associations that only become apparent after the onset of developmental windows in which significant synaptic pruning occurs in typically developing children. Given that developmental trajectories of synaptic pruning vary across brain regions^43^, one would predict that cortical morphometric associations to these traits would appear earliest in cortical areas that typically are first to undergo synaptic pruning. In such areas, youths with maturational deficits would be observed to have larger grey matter morphometrics due to their attenuated loss of grey matter. Importantly, adolescent neurodevelopmental studies suggest that significant synaptic pruning is likely to be occurring between the preteen and teenage years^43^.

Although work with non-human animals suggests the neuroanatomy of reactive and proactive aggression is partially dissociable^35^, the evidence for such a neuroanatomical dissociation in humans, and especially in adolescents, is relatively sparse. Thus far, structural investigations suggest that regions within the prefrontal cortex including the middle frontal gyrus, superior frontal gyrus, and medial prefrontal cortex^24,36^ are associated with both proactive and reactive aggression in adolescents. However, there is little consistency with respect to specific cortical morphometrics being associated with particular aggression subtypes. Specifically, while some authors have found negative associations between vlPFC grey matter and reactive aggression^36^, others have found this with respect to proactive aggression^24^. Notably, the neuroanatomical structures implicated in these aggression subtypes largely overlap with those that display inconsistent findings with respect to CU traits (e.g., medial prefrontal cortex and vlPFC). This raises the possibility that, like CU traits, the strength and direction of these associations may change across development. However, to the best of our knowledge, and despite considerable evidence suggesting prefrontal maturational abnormalities for related forms of behavioural dysregulation, this is the first study to investigate such cortical maturational patterns with respect to proactive and reactive aggression.

The primary objective of the current study was to examine how prefrontal cortical grey matter morphometrics relate to CU traits, proactive aggression, reactive aggression, and total aggression in a diverse sample comprised of youths both with and without disruptive behavioural disorders. In addition, we sought to examine how this relationship between prefrontal cortical morphometry and CU traits/aggression varied as a function of age (i.e., how age moderated this relationship). This investigation was motivated by three important observations. First, there is a lack of clarity in the direction of effects in studies examining the relationship between grey matter structure and CU traits/aggression. Second, there is a relative lack of data examining unique structural associations to proactive and reactive subtypes of aggression. Third, some of the inconsistent findings could suggest that CU traits and aggression may be associated with abnormalities in cortical maturation such that the nature of these associations varies across development (e.g.,^19,40,42^), as has been observed for other disorders featuring emotional or behavioural dysregulation^38,39^. Anatomically, these developmental changes are most likely observed in the prefrontal cortex given that neuroplasticity is particularly high in this region during adolescence^44^, and given other work indicating localized maturational abnormalities in the ACC, medial prefrontal cortex, and lateral prefrontal cortex^19,42^. In light of these findings, we explored these relationships with respect to the ACC, mOFC, and vlPFC. Given the evidence that CU traits/aggression may be associated with developmental aberrancies, we maintain that any structural association to these traits ought to change across development. However, based on the balance of evidence reviewed^17,18,20,21^, we expected cortical morphometrics within these ROIs to be negatively associated with CU traits/aggression. Furthermore, we expected age to significantly moderate the associations between prefrontal grey matter morphometrics and CU traits/aggression. Building on evidence suggesting that neurodevelopmental delays are associated with constructs and diagnoses related to CU traits/aggression, we expected this moderation effect to be positive (i.e., the correlation becomes more positive with increasing age, due to a possible synaptic pruning deficit). For both the correlation and moderation analyses, due to the lack of human evidence suggesting otherwise, we expected similar associations for reactive, proactive, and total aggression. While some neurocognitive models place a greater emphasis on vlPFC areas for regulating reactive aggression^30^, vlPFC has also been linked to emotional empathy which is thought to modulate risk for proactive aggression^34^. The current design will allow us to clarify these works and determine whether vlPFC morphometrics and development relate differentially to reactive and proactive aggression.

## Results

### Correlation analyses

See Table 1 for a full summary of the relationships between cortical morphometrics and both CU traits and aggression. Below, we discuss only those effects that survived correction for multiple comparisons after controlling for IQ and sex as nuisance variables.

**Table 1:** Correlational analyses investigating the relationships between cortical morphometrics and both CU traits and aggression.

	CU traits		Proactive aggression		Reactive aggression		Total aggression	
	*r*	*p*	*r*	*p*	*r*	*p*	*r*	*p*
Volume								
ACC	0.358	0.009***	0.172	0.222	0.185	0.190	0.191	0.174
mOFC	0.023	0.869	− 0.123	0.387	− 0.033	0.814	− 0.079	0.576
vlPFC	0.263	0.060	− 0.002	0.988	0.168	0.233	0.078	0.583
Surface area								
ACC	0.371	0.007***	0.070	0.624	0.177	0.210	0.115	0.417
mOFC	0.092	0.516	− 0.184	0.191	0.022	0.879	− 0.108	0.447
vlPFC	0.183	0.193	− 0.076	0.590	0.114	0.422	− 0.007	0.959
Thickness								
ACC	0.019	0.894	0.025	0.862	− 0.105	0.459	− 0.042	0.765
mOFC	− 0.199	0.157	− 0.226	0.108	− 0.264	0.059	− 0.263	0.060
vlPFC	0.207	0.142	0.079	0.578	0.188	0.183	0.149	0.291

*CU *callous-unemotional,* ACC *anterior cingulate cortex,* mOFC *medial orbitofrontal cortex,* vlPFC *ventrolateral prefrontal cortex*.*

*Denotes significance (*p* < 0.05).

***Denotes significance after correction for multiple comparisons.

#### CU traits

**Figure 1:**
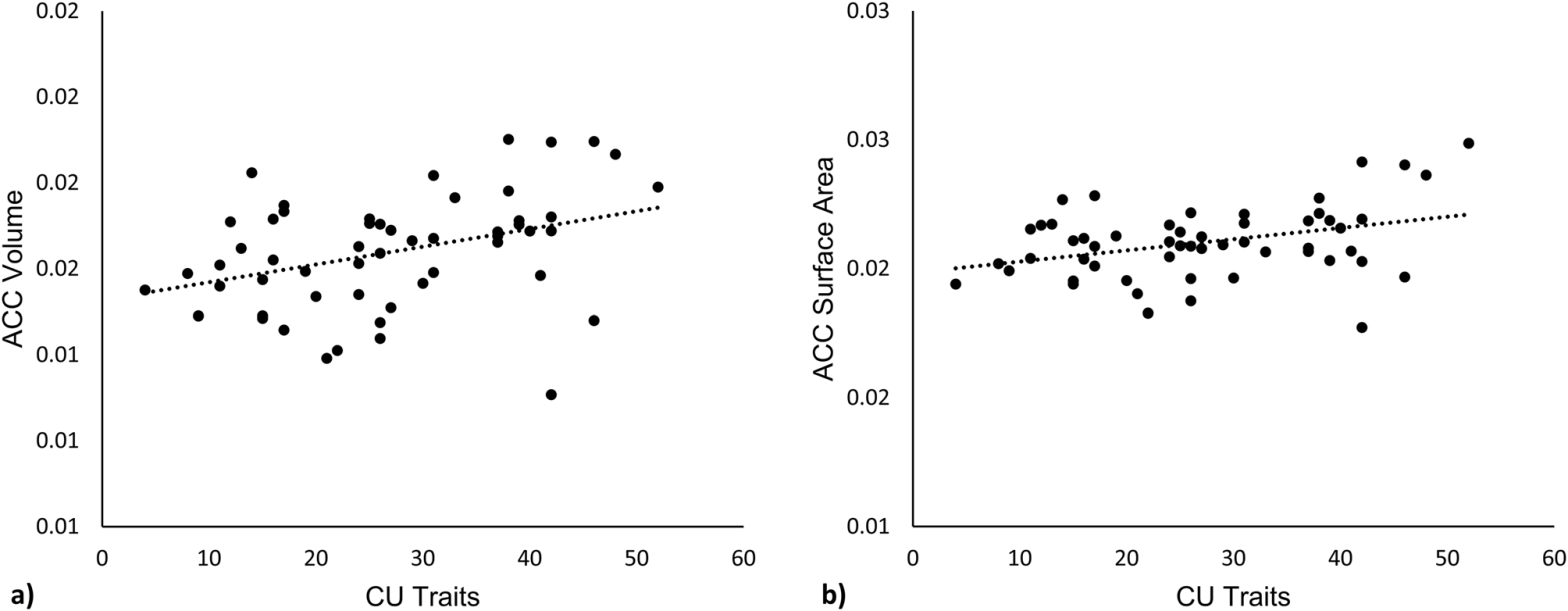
Neurostructural correlates of CU traits. Neurostructural relationships between (**a**) ACC volume and CU traits, and (**b**) ACC surface area and CU traits. Morphometrics are expressed as a fraction of whole-brain values. *CU *callous-unemotional,* ACC *anterior cingulate cortex*.*

First, we investigated the relationship between cortical morphometrics and CU traits. With respect to cortical volume, bilateral ACC volume was significantly positively associated with CU traits (*r* = 0.358, *p* = 0.009; Fig. 1a). Similarly, bilateral ACC surface area was positively associated with CU traits (*r* = 0.371, *p* = 0.007; Fig. 1b). There were no significant relationships between cortical thickness and CU traits.

#### Aggression measures

No relationships between cortical morphometrics and aggression measures survived correction for multiple comparisons.

### Moderation analyses

See Table 2 for a full summary of the moderation analysis results pertaining to CU traits, and Table 3 as it relates to aggression. Moderation analyses were conducted while controlling for IQ and sex as nuisance variables.

**Table 2:** Regression analyses investigating age as a moderating variable in the relationship between cortical morphometrics and CU traits.

	Volume				Surface area				Thickness			
	β	B	SEB	*p*	β	B	SEB	*p*	β	B	SEB	*p*
ACC	0.22	921.68	560.36	0.11	0.19	694.65	464.93	0.14	− 0.11	− 23.82	31.02	0.45
mOFC	0.32	1535.93	597.30	0.01***	0.26	791.64	438.94	0.08	− 0.01	− 2.21	25.75	0.93
vlPFC	0.08	278.82	476.91	0.56	0.12	315.93	362.36	0.39	0.08	21.84	39.50	0.58

*ACC* anterior cingulate cortex,* mOFC *medial orbitofrontal cortex,* vlPFC *ventrolateral prefrontal cortex.

*Denotes *p* < 0.05.

***Denotes significance after correction for multiple comparisons.

**Table 3:** Regression analyses investigating age as a moderating variable in the relationship between cortical morphometrics and proactive aggression, reactive aggression, and total aggression.

	Proactive aggression				Reactive aggression				Total aggression			
	β	B	SEB	*p*	β	B	SEB	*p*	β	B	SEB	*p*
Volume												
ACC	0.07	117.21	255.40	0.65	0.19	257.75	204.68	0.21	0.11	402.68	521.99	0.44
mOFC	0.24	479.41	255.85	0.07	0.21	338.07	212.82	0.12	0.25	1036.44	528.20	0.06
vlPFC	0.15	210.25	206.64	0.31	0.20	221.34	164.53	0.19	0.17	475.90	422.69	0.27
Surface area												
ACC	0.15	230.07	212.56	0.28	0.24	280.38	168.16	0.10	0.18	532.53	433.94	0.23
mOFC	0.32	405.09	179.09	0.03*	0.37	380.28	146.23	0.01***	0.35	914.79	369.52	0.02*
vlPFC	0.21	240.43	152.41	0.12	0.22	200.72	123.16	0.11	0.21	493.77	314.24	0.12
Thickness												
ACC	− 0.05	− 5.04	13.11	0.70	− 0.14	− 10.07	10.52	0.34	− 0.06	− 11.23	26.90	0.68
mOFC	0.13	10.49	10.69	0.33	− 0.18	− 11.52	8.54	0.18	0.02	2.87	22.01	0.90
vlPFC	0.14	17.05	16.81	0.32	0.13	11.97	13.54	0.38	0.14	35.26	34.34	0.31

*ACC *anterior cingulate cortex,* mOFC *medial orbitofrontal cortex,* vlPFC *ventrolateral prefrontal cortex*.*

*Denotes *p* < 0.05.

***Denotes significance after correction for multiple comparisons.

#### CU traits

**Figure 2:**
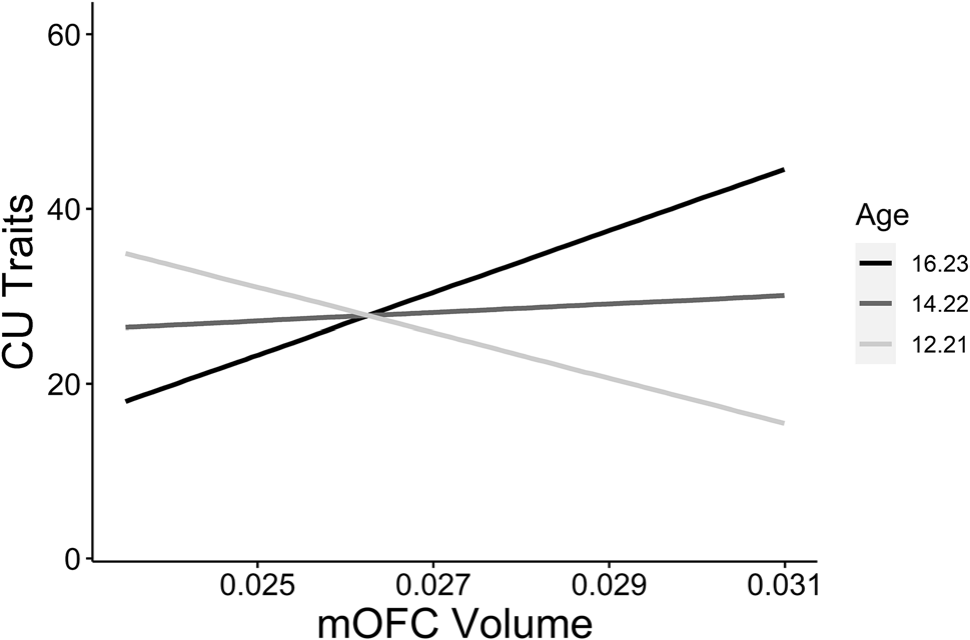
Age-specific neurostructural correlates of CU traits. Simple slopes analysis showing distinguishable associations between mOFC volume and CU traits at varying levels of the moderator variable age (1SD below mean age, mean age, 1SD above mean age). Morphometrics are expressed as a fraction of whole-brain values. *CU *callous-unemotional, *mOFC* medial orbitofrontal cortex*.*

First, we investigated age as a moderating variable in the relationships between cortical morphometrics and CU traits. Age moderated the relationship between bilateral mOFC volume and CU traits (β = 0.32, *p*_*moderation*_ = 0.013; Fig. 2). This moderation effect was such that the association between bilateral mOFC volume and CU traits became more positive with increasing age. Simple slopes analysis revealed that the conditional effect of mOFC volume on CU traits was significant and positive at 1SD above the mean age (β = 0.49, *p* = 0.031), less positive at the mean age (β = 0.07, *p* = 0.599), and negative at 1SD below the mean age (β = − 0.36, *p* = 0.069). Age did not significantly moderate any of the relationships between cortical surface area/thickness and CU traits.

#### Aggression measures

**Figure 3:**
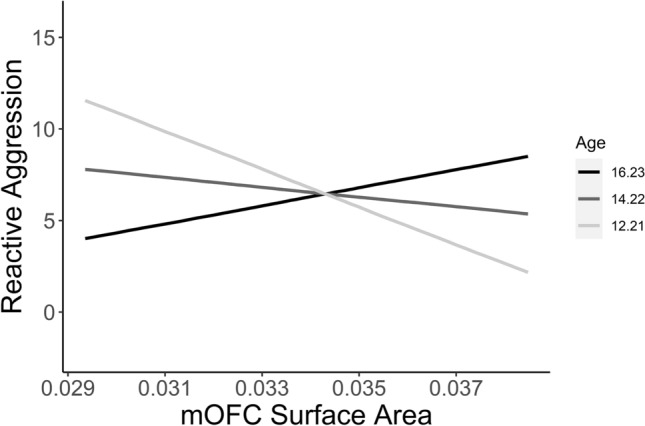
Age-specific neurostructural correlates of reactive aggression. Simple slopes analysis showing distinguishable associations between mOFC surface area and reactive aggression at varying levels of the moderator variable age (1SD below mean age, mean age, 1SD above mean age). Morphometrics are expressed as a fraction of whole-brain values. *CU *callous-unemotional, *mOFC *medial orbitofrontal cortex*.*

Age moderated the relationship between mOFC surface area and reactive aggression (β = 0.37, *p*_*moderation*_ = 0.012; Fig. 3). Simple slopes analysis revealed that the conditional effect of mOFC surface area on reactive aggression was positive at 1SD above the mean age (β = 0.24, *p* = 0.126), slightly negative at the mean age (β = − 0.13, *p* = 0.367), and significantly negative at 1SD below the mean age (β = − 0.49, *p* = 0.042). Age did not significantly moderate any of the relationships between cortical volume/thickness and proactive/reactive/total aggression.

## Discussion

Thus far, the literature has suggested that prefrontal structural abnormalities in the ACC, mOFC, and vlPFC are associated with elevated levels of CU traits^16–22^ and aggression^24,36^. However, there has been a lack of clarity in the direction of these associations. For instance, with respect to the ACC, youths with high CU traits have been found to have both reduced ACC grey matter volume^18^, increased ACC grey matter concentration^16^, and reduced developmental losses in grey matter volume^19^. Similarly, studies have found both negative^18,20,21^ and positive^16,24^ associations with CU traits/aggression for OFC volume and concentration. In the vlPFC and middle frontal gyrus, two areas with high anatomical proximity that are often used synonymously in the literature, there have been both negative^17,18^ and positive morphometric associations with CU traits^16,22^. In reviewing these conflicting data, it is important to note that the observed differences in the nature of these effects may be influenced by the instrument used to measure aggression/CU traits (e.g., self-report vs. parent-report vs. paradigm-based). However, regardless of the direction, this research and neurocognitive models^15,27,30^ have given us reason to believe that these prefrontal cortical structures are associated with CU traits and aggression. Accordingly, and in line with the balance of existing evidence^17,18,20,21^, we hypothesized that cortical morphometrics within the ACC, mOFC, and vlPFC would be negatively associated with CU traits and aggression. Contrary to our hypothesis, we found that ACC surface area and volume were positively associated with CU traits. We did not find any correlation between morphometrics of the mOFC or vlPFC with CU traits or aggression.

Past research has implicated lags in cortical maturation with disorders characterized by emotional/behavioural dysregulation such as ADHD^38,39^ and disruptive behavioural disorders^37^. However, there has been a comparative lack of research directly investigating this pattern of delayed cortical maturation in youths with elevated levels of CU traits and aggression. If a delay in cortical maturation is a feature seen in individuals with elevated CU traits/aggression, then we would expect the nature of the neurostructural associations to these traits to change with age. For example, a structural association may arise only when typically developing youths are undergoing significant synaptic pruning, and, due to their maturational delay, youths with elevated CU traits/aggression are not.

Applying the findings of these neurodevelopmental investigations of ADHD and disruptive behavioural disorders to transdiagnostic measures of CU traits and aggression, we expected that the neurostructural associations to these traits may vary as a function of age. In line with our predictions, age significantly moderated the relationship between mOFC volume and CU traits. Specifically, the association between mOFC volume and CU traits became more positive as age increased. With this same direction of moderation, age was a significant moderator in the relationship between mOFC surface area and reactive aggression. These more positive associations between morphometrics and CU traits/aggression in the older youths may reflect a reduction in the typical regressive cortical maturation (i.e., synaptic pruning) observed throughout adolescence. These positive associations may be absent in younger individuals because the neurotypical pattern of significant regressive cortical maturation has not yet occurred.

There were moderation effects at uncorrected or trend levels that may be noteworthy. Specifically, at an uncorrected level, there was evidence that age moderated the relationship between mOFC surface area and total aggression (β = 0.35, *p* = 0.017); the effect size of this pattern is considered medium^45^. Also not surviving correction for multiple comparisons, age moderated the relationship between mOFC surface area and proactive aggression (β = 0.32, *p* = 0.028); this association has a medium effect size^45^. At a trend level, age moderated numerous other relationships between cortical morphometrics and CU traits/aggression, including aggression subtypes (see Tables 2, 3). For all relationships between neurostructure and CU traits/aggression (and its subtypes) in which age was a moderator (at a significant, uncorrected, or trend level), the direction of moderation was consistent; as age increased, so too did the associations between neurostructure and aggression (for graphical illustrations of these effects, see Supplementary Fig. S1 online). This direction of moderation is consistent with the notion of a potential delay or deficit in cortical maturation being associated with heightened levels of CU traits/aggression in youths. To the best of our knowledge, no prior studies have investigated this developmental pattern with respect to proactive and reactive aggression. We hope this may serve to motivate future work to explore this pattern in larger samples.

The current results suggest that the degree to which specific prefrontal morphometric correlates to CU traits/aggression emerge may depend on age. Likely, the structural correlates of these traits are particularly apparent during periods of time in which typically developing youths tend to undergo significant cortical maturation. This would make a delay in such cortical maturation more evident. Extending this, and considering that the timing of neurodevelopment varies considerably across brain regions^43^, different brain regions may have unique timeframes during which they have reliable structural correlates to CU traits/aggression. We speculate that this abnormal cortical maturation may explain the inconsistent morphometric associations observed in the literature thus far.

As heterogeneity exists within the population of youths diagnosed with disruptive behavioural disorders^46^, and a subset of specific characteristics (e.g., CU traits and aggression) cross diagnostic boundaries, the present study took a transdiagnostic approach to examine how age may moderate the relationship between morphometric features and CU traits/aggression subtypes. To illustrate the heterogeneity associated with diagnostic categories, consider that while ODD, CD and ADHD are all classified as externalizing disorders^47^, there is a great deal of variability in the level of CU traits and the specific subtypes of aggression expressed by these youths both within and across diagnoses^48^. Nevertheless, transdiagnostic measures such as CU traits and proactive aggression help distinguish a group with particularly problematic outcomes^2,4,5,8^ that may require distinct interventions^12^. As a consequence, there is considerable interest in examining the unique neurobiological risk factors implicated in these features as they vary across diagnoses as well as within typically developing individuals^14,15^.

With a focus on CU traits and specific aggression subtypes, our study aimed to clarify the neurostucture associated with these emotional characteristics and problematic behavioural patterns. Our study builds on existing literature that has provided early evidence that CU traits and aggression are associated with cortical developmental abnormalities. For example, using the N200 event-related potential, which characteristically decreases in amplitude as the cortex matures^49^, Sumich et al.^40^ found that high levels of CU traits in youth were positively associated with N200 amplitude after covarying for age. This suggests that CU traits may be associated with deficient cortical maturation. These findings have since been corroborated with structural evidence; Tyborowska et al.^19^ found that longitudinal reductions in ACC grey matter volume between age 14 and 17 are negatively associated with CU traits (i.e., those with high CU traits experienced less developmental grey matter volume reductions). Compatible with these findings, higher levels of prosociality are associated with greater early-adolescent cortical thinning in neural regions involved in social cognition^42^. Furthermore, lower levels of prosociality are also associated with a slower, but prolonged course of cortical thinning that persists beyond adolescence and into the mid twenties^42^. This initially reduced rate but prolonged duration of cortical thinning may explain the inconsistent and often positive structural associations with CU traits in adolescence, and also the more consistent negative structural associations with psychopathic traits in adults (for review, see^50^). Our findings extend the previous literature by including, in one study, measures of CU traits, proactive, reactive, and total aggression as they relate to cortical volume, cortical surface area and cortical thickness. To the best of our knowledge, this is the first study to examine structural neurodevelopmental abnormalities associated with proactive and reactive aggression in adolescence. Further, we believe it is also the first study to use measures of cortical surface area while investigating the neurodevelopmental abnormalities associated with CU traits and aggression (of any subtype). The past literature has focused entirely on measures of cortical volume and thickness^19,37–39,41,42^. Importantly, developmental trajectories of cortical surface area appear to be at least partially independent of the trajectories associated with the other cortical morphometrics. That is, trajectories of cortical surface area are more dissociable from those of thickness and volume than the trajectories of thickness and volume are from each other^43^. As such, the inclusion of surface area measurements in our study provides a new perspective to the growing literature regarding the cortical maturational deficits associated with elevated levels of CU traits and aggression. Importantly, we found that age moderated the relationship between mOFC surface area and reactive aggression (β = 0.37, *p* = 0.012). Additionally, at uncorrected levels, age moderated the relationship between mOFC surface area and both proactive (β = 0.32, *p* = 0.028) and total aggression (β = 0.35, *p* = 0.017). As such, neurodevelopmental abnormalities potentially associated with aggression may be particularly evident in measures of mOFC surface area.

When interpreting the results of the current study, it is important to consider that CU traits and aggression are relatively stable in childhood and adolescence^51,52^. Despite this stability, we observed that the relationship between neural anatomical structure and these traits varied as a function of age. If such associations continue to be uncovered, they suggest that, in at least some regions of the prefrontal cortex, a specific cortical morphometric abnormality (e.g., reduced or increased grey matter) is unlikely to drive changes in CU traits and aggression. However, this does not exclude the possibility that factors related to cortical maturational deficits play an active role in the development of CU traits and aggression. Indeed, in mouse models, genetic alteration of loci encoding autophagy-related proteins which support microglia-dependent synaptic pruning leads to social behavioural deficits^53^.

To comprehensively discern the role of cortical maturation in the development of CU traits and aggression, future longitudinal data considering both changes in the expression of CU traits and aggression as well as developmental changes in neurostructural features are needed. Further clarification can be achieved by addressing which specific developmental processes underlie the structural developmental patterns associated with CU traits and aggression. While much of the existing literature and our discussion has focused on synaptic pruning, it is also possible that the negative associations that we observed between grey matter structure and CU traits/aggression in the younger children are reflective of deficits in synaptogenesis. Considering that the onset and cessation of synaptic pruning and synaptogenesis are thought to be spurred on by dissociable molecular mechanisms^54,55^, clarifying which developmental processes are aberrant in youths with high CU traits/aggression may allow future studies to generate specific hypotheses regarding the molecular features associated with these behavioural problems. Work of this nature may inform future targeted pharmacologic interventions that can be applied earlier in development. Such early interventions are likely to be particularly important for youth with CU traits/aggression as these features are developmental risk factors for adult psychopathy^9^, which has proven to be challenging to treat^10,11^.

It is important to note that our sample size is relatively modest, and results should be interpreted with caution until replicated in larger samples. However, we also note that the past literature uses samples of similar size^16,19,21,22^ and power analyses revealed that the study was adequately powered to detect medium effect sizes for both the correlational and moderation analyses. Nevertheless, it would be important to replicate these results in a larger sample, which would also allow for whole-brain analyses that might highlight neurodevelopmental abnormalities in other regions. Our results highlight the importance of considering the influence of developmental stage when interpreting the results of neurostructural findings.

In considering the influence of developmental stage, it is important to note that many neurodevelopmental changes associated with adolescence are spurred on by puberty, rather than chronological age^56^. Further, there is considerable variability in the age of pubertal onset^57^. As such, our study’s use of age as a proxy to measure neurodevelopmental status was indirect. Future studies should consider including pubertal stage as measured via a combination of scales and hormonal assays in the analysis^58^. It should also be noted that our sample contained very few female participants, which restricts the generalizability of the current results. It is critical that future work considers whether these associations are similar across male and female participants. Finally, whereas the current study relied on cross-sectional data, we hope it will help motivate larger-scale longitudinal investigations, which would allow for a more refined analysis concerning the regional and temporal specificity of these morphometric and developmental features.

## Conclusions

Our work suggests that CU traits/aggression in youth are related to abnormal trajectories of structural grey matter development. Specifically, we found that age moderated the relationships between mOFC volume and CU traits, and between mOFC surface area and reactive aggression. For all trend level or significant findings, the direction of the neurodevelopmental abnormality was consistent: in all cases, the association between grey matter structure and CU traits/aggression became more positive with age. This is in line with the notion of a cortical maturational deficit or delay, in which youths with elevated levels of CU traits and aggression undergo less or delayed regressive maturation in the form of synaptic pruning. This would manifest as increased grey matter being associated with CU traits and aggression in later adolescence. To build on these findings, future work should determine which developmental processes are impaired, in which regions, in which developmental timeframes, and what molecular processes underlie these developmental abnormalities. Ultimately, work of this nature may lead to the generation of interventions that target the neurodevelopmental factors that contribute to the development of problematic levels of CU traits/aggression.

## Methods

### Participants

Fifty-four children and adolescents aged 10–19 (7 females; 47 males) participated in this study. Participants were recruited from the community (via fliers and ads), youth treatment centers, and referrals from mental health professionals. Informed consent and assent were obtained from all youths and their parents. Because the primary interest in the study was dimensional measures of CU traits and aggression, the analyses were conducted trans-diagnostically. However, to characterize the sample, diagnoses were made using the Schedule for Affective Disorders and Schizophrenia for School-Age Children (K-SADS), which was administered by a trained team member (D.G.V.M) with the parent (a subset included both parent and child). Accordingly, the sample was comprised of 24 youths with either ODD (n = 20), disruptive mood disorder (n = 1) or CD (n = 3), 6 youths with attention deficit/hyperactivity disorder (ADHD) only, and 24 healthy controls. In the ODD/CD group, two met criteria for substance use disorder (cannabis), and 16 met criteria for ADHD. The study was approved by Western’s Health Sciences Research Ethics Board, and performed in accordance with the *Declaration of Helsinki*, Canada’s *Tri-Council Policy Statement,* and the *Uniform Requirements for manuscripts submitted to Biomedical journals*.

Exclusion criteria were estimated Intelligence Quotient (IQ) < 70, current diagnosis of psychosis, eating disorder, Tourette’s syndrome, autism spectrum disorder, mania, neurological disorders, or past severe head injury (loss of consciousness). Estimated IQ was evaluated using the Vocabulary and Matrix Reasoning subtests of the Weschler Abbreviated Scale of Intelligence (WASI-II). Groups differed on IQ (F = 7.696, *p* = 0.001). Specifically, the ODD/CD group had significantly lower IQ scores than the controls (*t* = − 3.93, *p* < 0.001), but did not differ from the ADHD-only group (*t* = − 0.214, *p* = 0.832). The IQ of the ADHD-only was lower than that of controls (*t* = − 2.24, *p* = 0.034). Groups did not differ on age (F = 1.103, *p* = 0.340). No groups differed significantly from the entire sample on sex ratio (χ^2^ = 1.190, *p* = 0.552). All demographic and clinical characteristics of the sample are reported in Table 4.

**Table 4:** Demographic and clinical characteristics of our sample.

Measure	Sample mean (SD)		
	ODD/CD (n = 24)	ADHD-only (n = 6)	Control (n = 24)
Age, years	14.05 (1.88)	15.36 (2.33)	14.10 (2.05)
Sex ratio, male:female	20:4	6:0	21:3
Estimated IQ	97.00 (12.05)	98.00 (15.67)	110.00 (10.73)
Medication stimulant	9	3	0
SSRI/SNRI	3	2	0
Atypical antipsychotic	2	1	0
Alpha agonist	2	0	0
Thyroid hormone	0	0	1
Proton pump inhibitors	0	0	1
Proactive aggression	8.92 (4.45)	1.33 (2.16)	1.79 (2.25)
Reactive aggression	10.17 (2.26)	3.83 (1.94)	3.71 (2.33)
Total aggression	23.42 (7.33)	5.67 (3.20)	7.50 (4.83)
CU traits	35.67 (9.15)	21.67 (6.80)	19.88 (9.63)

*IQ* intelligence quotient, *SSRI* selective serotonin reuptake inhibitors, *SNRI* selective norepinephrine reuptake inhibitors, *CU* callous-unemotional, *ODD* oppositional defiant disorder, *CD* conduct disorder, *ADHD* attention deficit/hyperactivity disorder.

*ODD/CD group has significantly higher proactive aggression, reactive aggression, total aggression, and CU traits than each of the other groups.

### Study measures

#### The Inventory of Callous-Unemotional Traits (ICU)

The ICU is a 24-item questionnaire that uses a 4-point Likert scale to assess levels of callousness, unemotionality, and uncaringness in youths^59^. Greater scores indicate higher levels of CU traits. While both parent- and self-report data were collected, only the parent-reported scores were used in the analyses as the parent-report is more predictive of future delinquency^60^, and has higher reliability than self-report data^61^. While the ICU has three subscales, only the composite score was included in the analyses due to concerns about its factor structure^62^. The measure possessed adequate internal consistency in our sample (α = 0.89).

#### Aggressive Behaviours Rating Scale (ABRS)

The ABRS is a 28-item questionnaire that assesses the frequency of aggressive behaviours in youth using a 3-point Likert scale, providing scores for reactive aggression, proactive aggression, and total aggression^63^. It is a revision of the relatively more common 6-item scale developed by Dodge and Coie^26^, with 22 additional items derived from a review of existing scales. A higher score indicates greater levels of aggression. The ABRS was administered to parents of the youths. This questionnaire has been validated in youths of similar ages to those in our sample^63,64^, and is a reliable assessment of subtypes of aggression when given to parents^63,65^. The measure possessed adequate internal consistency in our sample (α = 0.85).

### Image acquisition

T1-weighted anatomical scans were acquired using a 3-Tesla Siemens magnetic resonance imaging (MRI) scanner with a 32-Channel head coil at Western University’s Centre for Metabolic Mapping. The scan covered the whole brain (repetition time 2300 ms; echo time 2.98 ms; field of view 24.0 cm; flip angle 9°; 192 slices; 1 mm^3^ isovoxels; 240 × 256 × 192 matrix).

### Imaging analysis

Anatomical scans were processed using FreeSurfer (version 7.1.1), using the “recon-all” processing stream to obtain measures of cortical thickness, surface area, and volume (see https://surfer.nmr.mgh.harvard.edu/^66^). Briefly, FreeSurfer’s processing pipeline includes motion correction, transformation to Talairach space, intensity normalization, skull stripping, smoothing, segmentation of subcortical and white matter structures, and delineation of the grey matter–white matter boundary. FreeSurfer uses tissue-specific spatial intensity gradients, rather than absolute voxel intensity, to delineate the boundary between grey matter and white matter. Surface-based cortical inflations are then constructed, and any topological defects formed in the resulting models are automatically corrected using methods outlined in prior work^67,68^. The sulcal and gyral features of all participants are aligned to a common space such that their cortical geometry can be easily compared for accurate between-subjects comparisons.

Freesurfer segments the brain into distinct regions based on gyral/sulcal structure with a high degree of concordance to manual separation of the brain in both adults^69^ and youth^70^. Using surface-based morphometry, the software then quantifies cortical thickness, surface area, and volume. FreeSurfer’s use of surface-based (as opposed to voxel-based) morphometry in parcellating the cortex allows it to exceed the voxel resolution of the original data and detect submillimeter differences in cortical metrics^71^. Cortical thickness is measured as the shortest difference between the pia mater and the grey matter–white matter boundary, measured separately for each cortical vertex; FreeSurfer’s cortical thickness measurements have been validated against histological analyses and manual measurements^72^. Similarly, established methods have been adopted for measuring both cortical surface area^73^ and cortical volume^74^. FreeSurfer measures subcortical volume using voxel-based morphometry; surface-based morphometry cannot be applied subcortically due to the lack of an outwardly accessible white-matter surface precluding the formation of a surface-based reconstruction^66^.

We used FreeSurfer’s Destrieux atlas to parcellate the cortex. Table 5 shows how our ROIs were constructed as sums of raw FreeSurfer outputs.

**Table 5:** Regions of interest expressed as the sums of raw FreeSurfer outputs.

ROI	Atlas	FreeSurfer outputs
ACC	Destrieux	G_and_S_cingulAnt
mOFC	Destrieux	G_and_S_frontomargin + G_and_S_transv_frontopol + G_rectus + G_subcallosal + S_suborbital
vlPFC	Destrieux	G_front_inf-Opercular + G_front_inf-Orbital + G_front_inf-Triangul + S_orbital-lateral + S_orbital-H_Shaped

*vlPFC * ventrolateral prefrontal cortex,* mOFC * medial orbitofrontal cortex,* ACC * anterior cingulate cortex*.*

All regions of interest were investigated bilaterally, calculated as the sum of hemispheric volume and surface area values, and as the average of hemispheric thickness values. Resulting bilateral regional grey matter volumes were corrected for total grey matter volume to account for individual differences. This was done by dividing bilateral regional grey matter volume by total grey matter volume. Data were corrected to total grey matter volume, as opposed to intracranial volume or total brain volume, because total grey matter volume correlated least with age (*r* = − 0.03) and therefore did not account for the age-related differences in brain correlates to CU traits/aggression that we sought to investigate (see^75^ for a similar approach and rationale). Cortical grey matter thickness and surface area data were corrected in a similar fashion for mean cortical thickness and total cortical surface area, respectively. Visual inspections of the FreeSurfer outputs were also performed, blinded to diagnosis and measures of CU traits and aggression. No atypical errors were detected, and as such no participants were excluded on this basis^76^.

### Statistical analyses

There were no univariate or bivariate outliers in the questionnaire or neuroimaging data, defined respectively as data points greater than three standard deviations above or below the mean or with Mahalanobis values of *p* < 0.001.

Correlational analyses were conducted in Statistical Package for the Social Sciences (SPSS) version 28.0.1.1 to test the hypothesis that there would be distinct neurostructural correlates of CU traits and aggression in our sample. Specifically, we examined the bivariate correlations between the volume, surface area, and thickness of the ACC, mOFC, and vlPFC with questionnaire measures of CU traits, proactive aggression, reactive aggression, and total aggression. The analyses were conducted while controlling for IQ and sex as covariates. While total brain gray matter morphometrics were not included as a covariate, between-subjects differences in individual total brain grey matter volume were accounted for in all analyses by dividing regional morphometrics by total brain morphometrics (e.g., bilateral mOFC grey matter volume by total brain grey matter volume), as validated in past literature (e.g.,^77,78^). For this analysis, sample sizes were validated based on effect size estimates from previous work using similar ROIs and analytic techniques^18^. Based on the medium effect size in this study, we calculated that our sample size of 54 yields a power greater than 0.80. As such, our sample of 54 is adequately powered for this analysis.

A moderation analysis was conducted in Statistical Package for the Social Sciences (SPSS) version 28.0.1.1 to test the prediction that age alters the nature of the association between brain structure and CU traits and aggression. Specifically, we investigated age as a moderating variable in the relationships between the volume, surface area, and thickness of the ACC, mOFC, and vlPFC and proactive aggression, reactive aggression, total aggression, and CU traits. For each model, the dependent variable was the behavioural measure (e.g., aggression measure or CU traits), and the predictor variables included were a mean centered cortical morphometric of one of our ROIs, mean centered participant age, an interaction term (mean centered cortical morphometric × mean centered age), participant sex, and participant IQ. The latter two were entered as covariates to control for the impact they may have on the neurostructural association we sought to investigate. Mean centering of the variables comprising the interaction term was performed to ease data interpretation and mitigate potential issues related to multicollinearity^79^. Significant interactions were clarified using simple slopes analysis to estimate the conditional effects of brain structure on CU traits/aggression at various levels of the moderator variable age (1 SD below mean age, mean age, 1 SD above mean age). The simple slopes analyses were performed using the SPSS Process Macro. Due to the scarcity of research investigating age as a moderator in the relationship between brain structure and CU traits/aggression, a power analysis was conducted for this analysis using one effect size from a study similar to ours^16^. Given the medium effect size observed in their moderation analysis, we calculated that our sample size of 54 yields a power greater than 0.85. As such, our sample of 54 is adequately powered for this analysis.

At the request of anonymous reviewers, we performed additional analyses investigating the structural correlates to CU traits/aggression including ones that: (a) controlled for other behavioural measures (e.g., correlates to proactive aggression while controlling for reactive aggression); (b) used age as a quadratic (rather than a linear) moderator in the relationships between neurostructure and CU traits/aggression; (c) explored the structure of the nucleus accumbens, amygdala, and anterior insula in the development of CU traits/aggression. Results for these analyses can be found in the supplement (see Supplementary Tables S1–S5).

All data were corrected for multiple comparisons using a two-stage adaptive linear step-up procedure to control the false discovery rate (FDR)^80^. The corrections were run separately for the three structural (volume, surface area, thickness) measures and for each outcome measure (i.e., proactive aggression, reactive aggression, total aggression, and CU traits). For example, the cortical surface area correlates of proactive aggression were treated as one group separately from the cortical surface area correlates of reactive aggression. The FDR was 0.05.

### Supplementary Information

Supplementary Information.

## Data Availability

The data from which the findings of this study were derived are available from the corresponding author upon request.
