# Supplementary material for: Prefrontal cortex structural and developmental associations with callous-unemotional traits and aggression
Source: Sci Rep. 2024 Feb 19;14:4087. doi: 10.1038/s41598-024-54481-3 (PMC10876571; doi:10.1038/s41598-024-54481-3)
Supplement: Supplementary file 1 — Supplementary Information. [file 41598_2024_54481_MOESM1_ESM.pdf]

Title: Prefrontal cortex structural and developmental associations with callous-unemotional traits and aggression

Authors: Nathan Hostetler, Tamara P. Tavares, Mary B. Ritchie, Lindsay D. Oliver, Vanessa V. Chen, Steven Greening, Elizabeth C. Finger, and Derek G. V. Mitchell

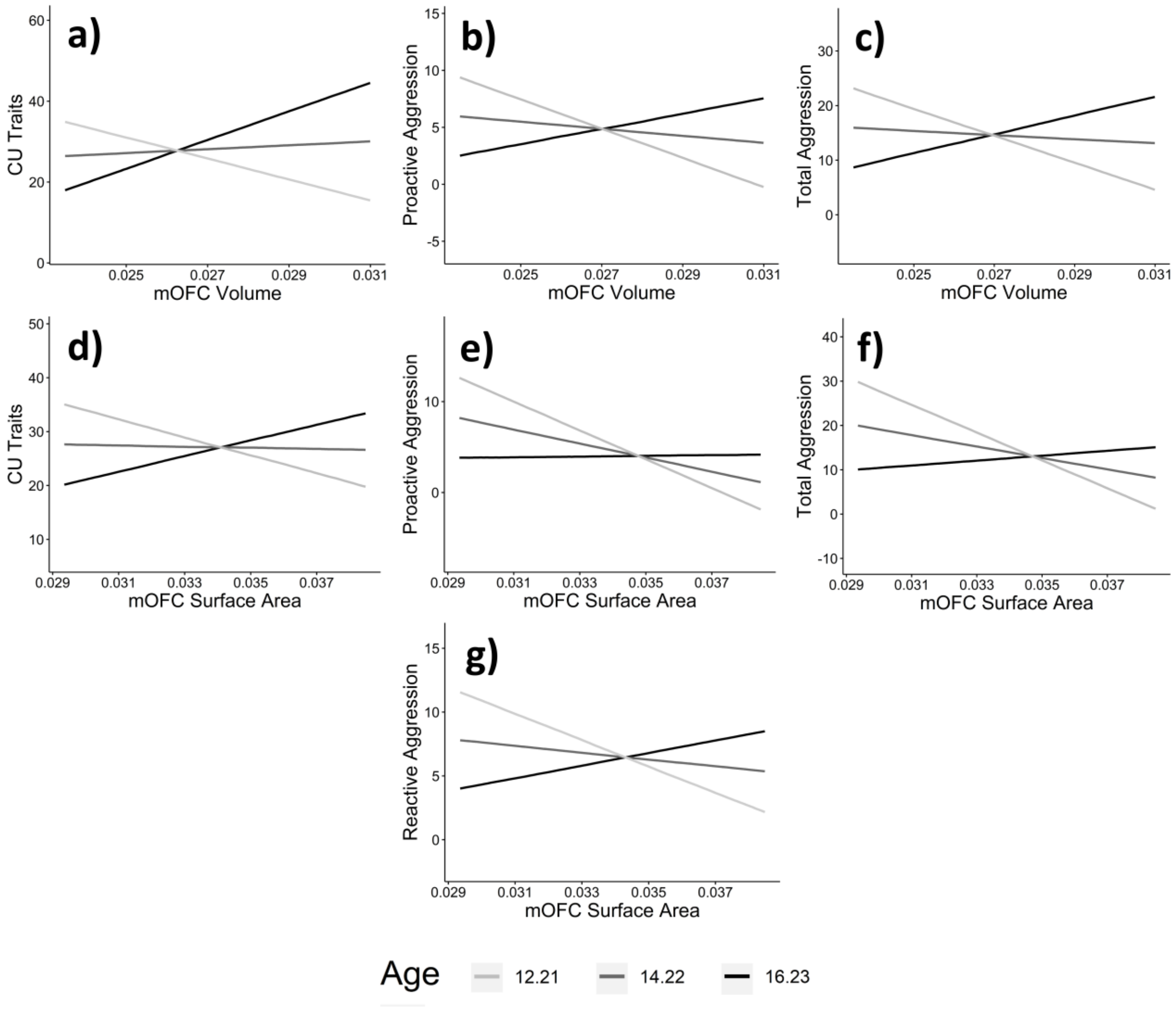

Supplementary Figure S1. Age-specific neurostructural correlates of CU traits and aggression subtypes.

Simple slopes analyses displaying the conditional effects of cortical morphometrics on CU traits/aggression (and its subtypes) at varying levels of the moderating variable age (1SD below mean age, mean age, 1SD above mean age). a) mOFC volume and CU traits (β = 0.32, *p* = 0.01), b) mOFC volume and proactive aggression (β = 0.24, *p* = 0.07), c) mOFC volume and total aggression (β = 0.25, *p* = 0.06), d) mOFC surface area and CU traits (β = 0.26, *p* = 0.08), e) mOFC surface area and proactive aggression (β = 0.32, *p* = 0.03), f) mOFC surface area and total aggression (β = 0.35, *p* = 0.02), g) mOFC surface area and reactive aggression (β = 0.37, *p* = 0.01).

Abbreviations: CU, callous-unemotional; mOFC, medial orbitofrontal cortex

Supplementary Table S1. Correlation analyses investigating the association between aggression (and its subtypes) and cortical morphometrics in the anterior insula, amygdala, and nucleus accumbens.

**VOLUME**	**Proactive Aggression**		**Reactive Aggression**		**Total Aggression**	
	r	*p*	*r*	*p*	*r*	*p*
aIns	0.062	0.661	0.111	0.433	0.096	0.496
Amygdala	-0.072	0.614	0.039	0.783	-0.005	0.974
nAcc	0.116	0.411	0.173	0.220	0.135	0.340
**SURFACE AREA**	**Proactive Aggression**		**Reactive Aggression**		**Total Aggression**	
	r	*p*	*r*	*p*	*r*	*p*
aIns	0.029	0.838	0.108	0.447	0.080	0.571
**THICKNESS**	**Proactive Aggression**		**Reactive Aggression**		**Total Aggression**	
	r	*p*	*r*	*p*	*r*	*p*
aIns	0.059	0.676	-0.061	0.665	-0.015	0.918

Abbreviations: aIns, anterior insula; nAcc, nucleus accumbens.
*Denotes $p < 0.05$.
***Denotes significance after correction for multiple comparisons.

Supplementary Table S2. Regression analyses investigating age as a moderating variable in the relationship between aggression (and its subtypes) and cortical morphometrics within the anterior insula, amygdala, and nucleus accumbens.

**VOLUME**	Proactive Aggression				Reactive Aggression				Total Aggression			
	β	B	SEB	*p*	β	B	SEB	*p*	β	B	SEB	*p*
aIns	0.00	7.17	241.39	0.98	0.13	178.35	194.26	0.36	0.05	195.28	494.70	0.70
Amygdala	0.23	1133.85	655.20	0.09	0.08	309.42	548.15	0.58	0.18	1880.39	1364.13	0.17
nAcc	0.02	209.53	1214.53	0.86	-0.08	-561.53	981.12	0.57	-0.02	-423.15	2491.18	0.87
**SURFACE AREA**	Proactive Aggression				Reactive Aggression				Total Aggression			
	β	B	SEB	*p*	β	B	SEB	*p*	β	B	SEB	*p*
aIns	0.04	47.81	180.48	0.79	0.14	151.35	144.76	0.30	0.08	230.59	369.15	0.53
**THICKNESS**	Proactive Aggression				Reactive Aggression				Total Aggression			
	β	B	SEB	*p*	β	B	SEB	*p*	β	B	SEB	*p*
aIns	-0.19	-14.38	9.92	0.15	-0.14	-8.55	8.17	0.30	-0.20	-31.48	20.41	0.13

Abbreviations: aIns, anterior insula; nAcc, nucleus accumbens.
*Denotes $p < 0.05$.
***Denotes significance after correction for multiple comparisons.

Supplementary Table S3. Regression analyses investigating age as a quadratic moderating variable in the relationship between cortical morphometrics and callous-unemotional traits.

	Volume				Surface Area				Thickness			
	β	B	SEB	*p*	β	B	SEB	*p*	β	B	SEB	*p*
ACC	-0.05	-105.94	265.87	0.69	-0.03	-52.21	242.49	0.83	-0.07	-5.51	16.40	0.74
mOFC	0.06	96.57	327.99	0.77	0.02	30.41	238.89	0.90	-0.18	-13.96	13.13	0.29
vlPFC	-0.09	-104.66	242.38	0.67	0.11	109.27	178.61	0.54	-0.01	-1.57	18.54	0.93

Abbreviations: ACC, anterior cingulate cortex; mOFC, medial orbitofrontal cortex; vlPFC, ventrolateral prefrontal cortex
*Denotes $p < 0.05$.
***Denotes significance after correction for multiple comparisons.

Supplementary Table S4. Regression analyses investigating age as a quadratic moderating variable in the relationship between cortical morphometrics and proactive aggression, reactive aggression, and total aggression.

**VOLUME**	Proactive Aggression				Reactive Aggression				Total Aggression			
	β	B	SEB	*p*	β	B	SEB	*p*	β	B	SEB	*p*
ACC	0.21	169.15	119.05	0.16	0.03	21.56	97.29	0.83	0.11	182.88	247.17	0.46
mOFC	0.08	51.97	143.07	0.72	0.18	91.23	118.49	0.45	0.08	109.58	295.22	0.71
vlPFC	0.30	151.04	103.77	0.15	0.23	94.17	83.32	0.26	0.26	275.33	213.28	0.20
aIns	0.36	310.67	117.92	0.01*	0.04	25.07	101.73	0.81	0.23	405.18	252.25	0.12
Amygdala	-0.07	-162.54	308.46	0.60	0.13	230.68	259.51	0.38	0.02	75.00	645.94	0.91
nAcc	-0.24	-1133.82	602.67	0.07	-0.13	-492.90	498.80	0.33	-0.19	-1798.92	1256.30	0.16
**SURFACE AREA**	Proactive Aggression				Reactive Aggression				Total Aggression			
	β	B	SEB	*p*	β	B	SEB	*p*	β	B	SEB	*p*
ACC	0.19	141.00	109.94	0.21	-0.01	-7.00	88.60	0.94	0.89	136.71	227.50	0.55
mOFC	-0.02	-11.90	100.92	0.91	0.20	88.90	81.16	0.28	0.07	87.16	207.18	0.68
vlPFC	0.26	110.52	74.87	0.15	0.35	117.47	59.54	0.05	0.31	268.46	152.84	0.09
aIns	0.54	345.64	92.11	<0.001***	0.22	109.86	82.89	0.19	0.43	561.63	198.68	0.01***
**THICKNESS**	Proactive Aggression				Reactive Aggression				Total Aggression			
	β	B	SEB	*p*	β	B	SEB	*p*	β	B	SEB	*p*
ACC	0.06	1.97	7.08	0.78	0.34	8.58	5.54	0.13	0.18	11.77	14.42	0.42
mOFC	-0.05	-1.48	5.59	0.79	-0.16	-4.21	4.43	0.35	-0.10	-6.45	11.48	0.58
vlPFC	0.02	1.00	8.01	0.90	0.12	4.14	6.43	0.52	0.05	4.25	16.34	0.80
aIns	0.01	0.16	4.46	0.97	-0.12	-2.98	3.65	0.42	-0.06	-4.15	9.16	0.65

Abbreviations: ACC, anterior cingulate cortex; mOFC, medial orbitofrontal cortex; vlPFC, ventrolateral prefrontal cortex; aIns, anterior insula; nAcc, nucleus accumbens.
*Denotes *p* < 0.05.
***Denotes significance after correction for multiple comparisons.

Supplementary Table S5. Correlation analyses investigating the relationship between cortical morphometrics and both proactive aggression and reactive aggression, while controlling for other emotional/behavioural features of interest.

**VOLUME**	**Proactive Aggression**		**Reactive Aggression**		**Reactive Aggression**	
**Controlling for:**	**Reactive Aggression**		**Proactive Aggression**		**CU Traits**	
	r	*p*	*r*	*p*	*r*	*p*
ACC	0.058	0.684	0.089	0.535	-0.091	0.523
mOFC	-0.141	0.322	0.079	0.584	-0.068	0.633
vlPFC	-0.179	0.210	0.244	0.085	-0.018	0.900
aIns	-0.025	0.862	0.096	0.505	-0.122	0.395
Amygdala	-0.143	0.317	0.130	0.363	-0.095	0.508
nAcc	-0.011	0.939	0.129	0.366	0.174	0.221
**SURFACE AREA**	**Proactive Aggression**		**Reactive Aggression**		**Reactive Aggression**	
**Controlling for:**	**Reactive Aggression**		**Proactive Aggression**		**CU Traits**	
	r	*p*	*r*	*p*	*r*	*p*
ACC	-0.083	0.561	0.182	0.200	-0.117	0.412
mOFC	-0.286	0.042*	0.224	0.114	-0.058	0.685
vlPFC	-0.228	0.108	0.242	0.087	-0.018	0.902
aIns	-0.069	0.629	0.125	0.384	-0.074	0.608
**THICKNESS**	**Proactive Aggression**		**Reactive Aggression**		**Reactive Aggression**	
**Controlling for:**	**Reactive Aggression**		**Proactive Aggression**		**CU Traits**	
	r	*p*	*r*	*p*	*r*	*p*
ACC	0.144	0.314	-0.176	0.217	-0.163	0.254
mOFC	-0.054	0.706	-0.150	0.292	-0.178	0.211
vlPFC	-0.081	0.573	0.188	0.186	0.064	0.656
aIns	0.149	0.298	-0.149	0.295	-0.224	0.115

Abbreviations: ACC, anterior cingulate cortex; mOFC, medial orbitofrontal cortex; vlPFC, ventrolateral prefrontal cortex; aIns, anterior insula; nAcc, nucleus accumbens.
*Denotes *p* < 0.05.
***Denotes significance after correction for multiple comparisons.
